# Crash course in EPaCCS (Electronic Palliative Care Coordination Systems): 8 years of successes and failures in patient data sharing to learn from

**DOI:** 10.1136/bmjspcare-2015-001059

**Published:** 2016-09-16

**Authors:** Mila Petrova, Julia Riley, Julian Abel, Stephen Barclay

**Affiliations:** 1 Primary Care Unit, Department of Public Health and Primary Care, University of Cambridge—Institute of Public Health, Cambridge, UK; 2 The Royal Marsden & Royal Brompton Palliative Care Service, London, UK; 3 Institute of Global Health Innovation, Imperial College, London, UK; 4 Weston Hospicecare and Weston Area Health Trust, Weston-super-Mare, UK

**Keywords:** electronic palliative care coordination systems, EPaCCS, Registries, Health Information Exchange, Electronic Health Records, Palliative Care

## Abstract

**Background:**

Electronic Palliative Care Coordination Systems (EPaCCS) are England's pre-eminent initiative in enabling advance care planning and improved communication and coordination at the end of life. EPaCCS have been under development for 8 years after being proposed, as Locality Registers, in the 2008 End of Life Care Strategy for England. EPaCCS are electronic registers or tools and processes for sharing data which aim to enable access to information about dying patients. Striking outcomes have been reported around EPaCCS, such as 77.8% of ‘Coordinate My Care’ patients dying in their preferred place. EPaCCS have, however, been extremely challenging to develop and implement, with many projects remaining continuously ‘under development’ or folding. They also continue to be suboptimally integrated with other data sharing initiatives. Rigorous research is non-existent.

**Discussion points:**

We discuss the current EPaCCS landscape and way forward. We summarise key facts concerning the availability, uptake, outcomes and costs of EPaCCS. We outline 5 key challenges (scope of projects, unrealistic expectations set by existing guidance, the discrepancy between IT realities in healthcare and our broader lives, information governance and ‘death register’ associations) and 6 key drivers (robust concept, striking outcomes, national support and strong clinical leadership, clinician commitment, education and funding).

**Conclusions:**

The priorities for advancing EPaCCS we propose include linking to other work streams and reframing the concept, potentially making it less ‘end of life’, overview of current EPaCCS and lessons learnt, continuing work on information standards, rethinking of national funding and new levels of individual and community involvement.

## Introduction

Advance care planning for the end of life is a responsibility of a growing number of healthcare professionals and an expanding niche for clinical and consumer IT products. Of the 10 leading causes of death worldwide, only one is a type of cancer, the historical priority of palliative care services.^1^ Non-communicable diseases accounted for 68% (38 million) of the world's deaths in 2012.^2^ End of life care is thus falling increasingly within the remit of chronic disease specialists. The specialised and often fragmented nature of healthcare in the developed world and the complexity of end of life needs already demand multiprofessional involvement: 36.1% of US Medicare beneficiaries were treated by 10 or more doctors in the last 6 months of life (2007 data).^3^ Generalist care, including out of hours, is also key: in a UK study of over 21 500 patients, general practitioners (GPs) were estimated to have recorded data on 7.3 days in the final 90 days of a patient's life.^4^ Online consumer products, such as My Directives (https://mydirectives.com), Best Endings (http://www.bestendings.com) and My Living Will (https://www.mylivingwill.org.uk), are also becoming popular.

We discuss England's pre-eminent initiative in enabling advance care planning and improved communication and coordination at the end of life—the Electronic Palliative Care Coordination Systems. EPaCCS are also a proof of concept for other conditions requiring multiprofessional and multisetting involvement, communication and coordination around expressed patients' wishes and a range of sources of patient data.

EPaCCS take various forms, which are still insufficiently documented—of web-based electronic registers, systems based on sharing care summaries and plans alongside patients' electronic records, patient portals, real-time extractions from the records of participating organisations, etc. They aim to provide up-to-date key information about patients believed to be in the last year of their life in GP practices, emergency telephone services (111 and 999), GP out of hours services, accident and emergency departments, ambulance services, hospitals, community nursing teams, specialist palliative care services, hospices and care homes. In the UK, each of these settings has their own (electronic or paper) patient record. EPaCCS aim to improve communication and coordination and ensure that all those involved in a patient's care are aware of their wishes, preferences and advance care plan. They are expected, and to an extent have been demonstrated, to enable more patients to die at their preferred place and reduce unnecessary hospital admissions and ambulance journeys, inappropriate interventions, use of unscheduled care and repeated ‘difficult conversations’.^5^ Provided that they have well-developed reporting functions, EPaCCS also supply detailed outcome metrics and enable continuous quality improvement in local end of life care services.

Striking outcomes have been reported around EPaCCS. For instance, 82.4% of the patients of ‘Coordinate My Care (CMC)’ have died outside of hospital; 77.8% died in their preferred place, with 71.8% of them dying in the place of their first preference (May 2016 data, since inception). Levels of hospital death in patients without cancer on and off the South West EPaCCS were found to be 8.3% vs 49.4%, respectively.^6^ Yet EPaCCS also face immense challenges, including ones of implementation, sustainability, cost-effectiveness, equality and service capacity.

EPaCCS take the idea of Locality Registers, proposed by the 2008 End of Life Care Strategy,^7^ into the age of digital communication. Evidence remains sparse. There are two evaluations of early implementers (2011 and 2013);^6^ ^8^ a recent national evaluation offering tentative conclusions, reflecting the substantial challenges of EPaCCS data collection and comparison;^9^ descriptive studies of CMC incorporating audit data;^10–13^ internal project reports,^14^ ^15^ only a small part of which are publicly available, and conference abstracts.^16–20^ The only completed peer-reviewed evaluation has substantial methodological limitations.^21^ Most evidence on EPaCCS—often anecdotal, at times overinterpreted by virtue of commitment to the initiative, and with limited information on methods and contexts—is solely accessible through policy documents and internal reports.

In terms of their multifunctionality and ambition, EPaCCS appear specific to the UK. They aim to coordinate care across all potential providers of care at the end of life; store a dynamic record of a patient's condition, treatment, wishes and preferences; enable advance care planning and accumulate data for service evaluation, quality improvement and research. Other countries have developed tools addressing some of these aims. Most notably, many maintain registers or databases on end of life care provision and outcomes aimed at quality assessment and improvement and research rather than care coordination. Examples include the Swedish National Quality Register in End of Life Care (The Swedish Register of Palliative Care, SRPC), which, as of 2015, contained data from two-thirds of all deaths in Sweden,^22^ ^23^ the Danish Palliative Database^24^ ^25^ and the PCOC (Palliative Care Outcomes Collaboration) of Australia (http://ahsri.uow.edu.au/pcoc/about/index.html). Transnational initiatives have also been tested, as in the EURO SENTIMELC study.^26^

In the USA, there is a growing use of electronic registries of Physician Orders for Life-Sustaining Treatment (POLST).^27–29^ POLST registries appear to focus entirely (Utah, Oregon)^27^ ^30^ or primarily (New York State)^31^ on patient preferences and wishes concerning treatment options such as resuscitation efforts or use of feeding tubes or intravenous fluids. In addition to providing a record of a patient's wishes and preferences, EPaCCS also contain a summary of their condition and care, medication information and details of carers and services involved, among others. POLST are legally binding, whereas most wishes and preferences recorded on EPaCCS are not. POLST registries thus tend to contain more legal fields, for instance concerning witness requirements, concurrent physician opinion and process of reaching a mental capacity decision.^31^

The Scottish electronic Palliative Care Summary (ePCS) is a central system updated two times per day from GP records, introduced in 2008 and fully rolled out by 2010.^32^ ^33^ An audit in Lothian found that 75% (year 2012) and 71% (year 2013) of specialist palliative care patients had electronic information available to out of hours services: a significant improvement relative to 49% in 2008.^32^ Ali *et al* 34 reported that not having an ePCS was associated with a higher risk of hospital admission (OR=2.43). ePCS have been regarded as a success and led to the development of a new Electronic Key Information Summary for patients with long-term conditions.^35^ England's EPaCCS and Scotland's ePCS are similar in the information covered and services involved, but also differ in substantial ways. For instance, ePCS is a centralised system, whereas EPaCCS are numerous and varied. A comparison of the two approaches is outstanding.

Two further developments are worth mentioning in contextualising EPaCCS: large-scale disease/treatment registers and health information exchange (HIE) initiatives. Many countries have well-established cancer registers. There is also a growing number of large-scale registers of other life-threatening conditions or treatments of these (eg, Switzerland's AMIS Plus national registry for patients with acute coronary syndrome and the joint Australia and New Zealand Dialysis and Transplant (ANZDATA) Registry).^36^ ^37^ Such registers perform some of the functions of EPaCCS, including patient identification and the generation of data for service evaluation, quality improvement and research. Finally, EPaCCS are a HIE initiative, as much as they have been discussed primarily in an end of life care context rather than healthcare IT. The number of HIE initiatives is rapidly growing. In the USA for instance, a 2012 survey found that 1398 hospitals (30%) and 23 341 ambulatory practices (10%) were participating in 119 operational HIE efforts, relative to 14% of hospitals, 3% of ambulatory practices and 75 operational HIE efforts 2 years earlier.^38^ A conceptual systematic review is needed to contextualise EPaCCS in the global availability of palliative and end of life registers, registers of life-threatening conditions and treatments for these and the broader HIE literature.

## Sources of data and discussion points

We present a critical analysis of EPaCCS against a background of scarce research, limited public awareness and insufficient openness in policy documents and the official discourse. We also offer an extensive structured framework for comparing the features, contexts and key outcomes of existing EPaCCS for use in future evaluations, benchmarking and funding decisions (see online supplementary tables: table S1 includes >60 parameters, table S2 lists the 51 items from the National Information Standard and 12 further fields). Finally, we make suggestions for the way forward, some of which represent a radical shift from what might be a natural trajectory of development.

10.1136/bmjspcare-2015-001059.supp1 Supplementary tables

The paper draws primarily on evidence and lessons from three projects: London's ‘Coordinate My Care (CMC)’, the South West EPaCCS and the Cambridgeshire & Peterborough Project for Data Sharing in End of Life Care (C&P Project). Online supplementary tables S1 and S2 summarise their characteristics. CMC is the largest EPaCCS in England, available to a population of over 9 million people. So far, 29 083 CMC urgent care plans have been created (May 2016 data, since inception, August 2010). It is also the system with the most extensive support infrastructure of all EPaCCS, including user support and training, information materials, clinical and information governance (IG) infrastructure, reporting and analysis work and high profile publicity. The South West EPaCCS is one of the earliest EPaCCS projects, initiated in 2008 before the national pilot (2009–2011)^8^ and later part of it. It also works in close collaboration with the National End of Life Care Intelligence Network (NEoLCIN) (http://www.endoflifecare-intelligence.org.uk/home). For a proportion of its users, the C&P Project is based on data sharing integrated with routine record keeping—the vision for the ‘ideal EPaCCS’, provided such integration can be achieved for all users.

Sources of data and discussion points include:

- Audit and evaluation data from internal monthly reports and public documents (CMC), existing evaluations (South West) and in-progress evaluations (C&P Project). References are given for published data; none come from peer-reviewed papers.
- Key costs data from the CMC and C&P Project teams.
- Data on features of the geographical areas covered: end of life care statistics from the NEoLCIN;^39^ data on population sizes and numbers of GP practices from NHS England;^40^ data on clinical services and teams obtained through Freedom of Information requests (9 for the South West, 1 for London, January–August 2015), key informants and team exercises in local service mapping.
- Descriptive EPaCCS data from the project leads and coauthors, organised around a framework developed for this paper (see online supplementary tables S1 and S2b) and the National Information Standard for EPaCCS (see online supplementary table S2a).
- Anecdotal evidence and discussion points concerning the drivers and challenges of the projects and the way forward from the project leads and coauthors. These were obtained through structured and unstructured email exchanges, interviews, meetings and telephone calls, the majority of which conducted between June and September 2014.
- The national EPaCCS Conferences in July 2015 and March 2016^41^ ^42^ and a critical review of policy documents were additional sources of data and considerations.

These data and discussion points were brought together by the first author in an extended internal report (available from the corresponding author), later distilled for this paper.

## Stage of development, outcomes and costs of EPaCCS

A 2013 survey suggested that work was underway on at least 82 EPaCCS—33 developed in partnerships involving 139 of the 211 Clinical Commissioning Groups (CCGs) in England and 49 independently.^43^ In November 2014, 91 (43%) CCGs reported that they had a functioning EPaCCS (or similar system), 53 (25%) had ‘plans’ and 2 (1%) had no plans for an EPaCCS. Progress was unknown in 65 (31%) CCGs.^44^

It is unclear how commonly used descriptors of ‘functioning’ and ‘operational’ relate to the usability and use of EPaCCS. A total of 26 249 patients were on the 18 systems (49 CCGs) for which data were provided in the 2013 survey.^43^ More up-to-date national data are not yet available although data collection for an EPaCCS Baseline Review 2015/2016 has been completed (to be published by NHS England in 2016/2017).^9^ Even established systems achieve far from optimal coverage: CMC, for instance, covers 16.6% of its estimated end of life population (December 2014).

Striking outcomes have been observed for EPaCCS patients, yet high quality evidence is lacking. Data from the South West on 3012 EPaCCS patients and over 67 000 total deaths demonstrated differences in hospital deaths of 9.8% vs 33.9% for patients with cancer and 8.3% vs 49.4% for patients without cancer, respectively, on and off EPaCCS.^6^ Of the CMC patients who died (12 362) between August 2010 and May 2016, 7614 had a preferred and actual place of death documented and 77.8% of these died in their preferred place (first preference achieved for 71.8%). An independent evaluation suggested average savings of £2100 per person who dies with a CMC care plan, through reductions in hospital attendance and length of stay aligned with patient wishes.^45^ My Care Choices, the Essex EPaCCS, reported that 43% of all deaths in their 38 participating practices were preidentified by the register and 86% of those patients were able to die in their preferred place.^20^ Estimates from the Nottinghamshire EPaCCS, concerning a caseload averaging 3345 patients, suggested a 45% lower rate of hospital admissions for EPaCCS patients (2.5% vs 4.5%).^14^ Data from the Bedfordshire EPaCCS show that 69.7% (235/337) of patients have not been conveyed to hospital after a contact between the ambulance service and the service hosting the EPaCCS.^46^ Those are impressive outcomes, but ones concerning potentially biased patient samples, affected by limited opportunities to control confounders reliably, representing evidence of association, not causation, and coming from a small range of successful projects with good reporting capabilities. The latest national evaluation^9^ was not able to identify a statistically significant difference between EPaCCS and non-EPaCCS sites in terms of improvements in death in usual place of residence, hospital admissions and resource use. An economic assessment could not be completed, largely due to limitations of the data that could be collected. Rigorous evaluation and research is urgently needed.

There is a concern that EPaCCS projects may be under-resourced. The 2013 Economic Evaluation uses as its default assumption costs of £21 104 for set-up and £8235 for annual maintenance, per 200 000 population per annum, with expectations of wider end of life care investment.^6^ While IT costs may be low with simpler solutions, those projects demand resources for securing stakeholder support, providing training and information, negotiating workflow and system changes, upgrading the EPaCCS, auditing and reporting, etc. Between August 2010 and April 2014, CMC cost ∼£1.5 m. Using the Economic Evaluation reference points, adjusting for a population size of 9.27 m and assuming 1.75 years have been dedicated to set-up and 2 years to annual maintenance, the projected cost of CMC is over £1.7 m for this time period. The C&P Project cost £245 000 between July 2012 and March 2016, whereas its estimated cost is £165 109 (adjusting for a population size of 0.86 m, and accounting for 20 months for set-up and 2.1 years for maintenance). While substantial progress has been made in local data sharing-in end of life care and more broadly-much further work remains to be undertaken.

## Key challenges

### Projects need to involve nine key service types and hundreds of individual settings

An EPaCCS project needs to involve, at an advanced though still imperfect level of uptake, GP practices, emergency telephone lines, ambulance services, GP out of hours services, hospitals, community nursing teams, specialist palliative care services, hospices and care homes. This translates into huge numbers of settings and teams, for example >330 for C&P, >1640 for the South West EPaCCS and >4600 for CMC (see online supplementary table S1). Most of these settings and teams have significant autonomy. There are no established levers for joint action of such a wide scope within a local health economy covered by an EPaCCS. An added challenge is that services with a broad coverage (eg, ambulance trusts) and/or close to geographical and health system boundaries will need to work with multiple EPaCCS.

### EPaCCS teams start a register project and find themselves transforming systems and culture

The nature and scope of EPaCCS projects is not apparent from existing guidance. When EPaCCS cover large areas and populations and have ambitious goals, they cannot be simply ‘end of life register’ projects. They become complex, long term, resource hungry initiatives that need to uncover and optimise existing care pathways, change workflows, patterns of collaboration and culture, educate health professionals and break new ground in data sharing.

### The realities of healthcare IT are far from the expectations we have from our daily IT lives

Clinical information systems in the UK are not interoperable, in spite of the enticing visions posing as round-the-corner realities in political speeches and in the marketing and advertising materials of IT companies. While mobile working is integral to our daily lives, paper records persist in many care settings.

Most EPaCCS developers have chosen one of two suboptimal solutions while working on or awaiting a new level of record integration: web-based solutions external to the record keeping system of any of the settings involved or ones internal to a locally dominant system. The former allow breadth of access and equality but require separate log-ins and double data entry, with the concomitant disruption of workflows, risks of error, out of date information and opportunity costs. The latter enable continuity with routine record keeping for users of the dominant system, but disadvantage users of other systems and, through this, the patients cared for by them. New generation solutions appear tantalisingly near—for example, the soon-to-be-launched My Right Care (http://www.myrightcare.co.uk) promises automatic population of a joint care plan from ‘all relevant services’ and an external, yet seamlessly integrated, interface but is still to pass the go-live challenges.

### IG-related decision-making for EPaCCS projects is not backed by a clear framework

The IG documents in box 1 add up to 629 pages, yet they do not address many of the IG issues associated with an EPaCCS. Some IG specialists concede that IG rules need to be ‘bent’ or controversially interpreted so that EPaCCS projects are not obstructed or abandoned, while the lawfulness of decisions is ensured within broader legal and governance frameworks.

Box 1 **Basic IG documents potentially relevant to EPaCCS developers**

*IG in EPaCCS*

Electronic Palliative Care Co-ordination Systems: Information Governance Guidance, January 2014, 20 pp.

http://systems.hscic.gov.uk/qipp/library/epaccsig.pdf

*Key documents from the broader IG framework*

Data Protection Act, 1998, 133 pp.

http://www.legislation.gov.uk/ukpga/1998/29/data.pdf

The Information Governance Review (Caldicott 2 Review), March 2013, 142 pp.

https://www.gov.uk/government/uploads/system/uploads/attachment_data/file/192572/2900774_InfoGovernance_accv2.pdf

Royal College of General Practitioners. Patient Online: The Road Map, March 2013, 63 pp.

http://elearning.rcgp.org.uk/mod/page/view.php?id=4709

Law Commission. Data Sharing between Public Bodies. A Scoping Report, July 2014, 179 pp.

https://www.gov.uk/government/publications/data-sharing-between-public-bodies-a-scoping-report

Information Commissioner's Office. Data Sharing Code of Practice, May 2011, 58 pp.

https://ico.org.uk/media/for-organisations/documents/1068/data_sharing_code_of_practice.pdf

*Key public facing documents*

The Care Record Guarantee, 15 pp.

http://systems.hscic.gov.uk/rasmartcards/documents/crg.pdf

Keeping your online health and social care records safe and secure (British Computer Society booklet for the general public), 19 pp.

http://www.nhs.uk/nhsengland/thenhs/records/healthrecords/documents/patientguidancebooklet.pdf

### End of life care is an emotive and uncertain domain

There are perceptions of EPaCCS as ‘death registers’. This is a significant obstacle to their uptake. It will be a death sentence if the association becomes widespread. A further challenge is the difficulty of prognosis in end of life care. For three of the four EPaCCS considered in the 2013 Economic Evaluation, the percentage of patients who had not died within a year was close to or higher than 50% (64.3%, 47.3%, 59.0%, estimates based on data presented in Table 3 of the Economic Evaluation).^6^ While these high percentages will partly be due to incomplete recording of deaths and inefficient deactivation of records, difficulties of prognosis in end of life care are likely to be a significant factor. Importantly, a substantial proportion of health professionals continue to experience difficulties in discussing death and having advance care planning conversations and/or have limited skills in identifying and managing patients at the end of their life.^47–50^ Box 2 outlines a number of further challenges faced by EPaCCS projects.

Box 2 **Further challenges to EPaCCS projects**

- Reporting and auditing difficulties:
  for EPaCCS which are not hosted by the team that developed them—complex processes and (perceptions of) IG constraints in having data released;
  for EPaCCS using a record sharing model—as there is no centralised register, a separate reporting solution is needed.
- Limited research and evaluation evidence.
- Challenges of effective education in a healthcare setting—with the limited availability of training time and difficulties of evaluating impact on outcomes.
- The skills set needed to lead and progress EPaCCS projects comes from too many and too different worlds; difficulties of communication between IT experts, end of life care specialists and managers.
- Broad context of initiative fatigue, and time and financial constraints.

## Key drivers

### Robust concept

The benefits of more and up-to-date information about dying patients can be grasped immediately. The concept has a fundamental solidity about it.

### Striking outcomes for patients on EPaCCS

Some of the outcomes for EPaCCS patients are impressive, as cited above.

### National support and strong clinical leadership

As locality registers, EPaCCS were proposed in the End of Life Care Strategy for England.^7^ The National End of Life Care Programme supported pilot work^8^ and led on their spread.^5^ Limited but reliable support has been available since the closure of the Programme from the NEoLCIN, NHS Improving Quality and national leads in end of life care. Recently, there has been a reinvigorated policy level interest in EPaCCS, as demonstrated by the commissioning of the national evaluation^9^ and still to be published Baseline Review.

Comparing the achievements of individual projects suggests that strong clinical leadership is another decisive factor for their success.

### Clinician commitment to end of life care

The recognition that we will all be facing end of life care issues, personally and in accompanying loved ones, and that we may have only ‘one chance to get it right’^51^ motivates many clinicians to provide exceptional service for their dying patients and those who care for them. Human kindness combined with high professionalism is the unshakeable foundation that sustains EPaCCS.

### End of life care education

Without attendant end of life care skills, limited uptake and superficial use of EPaCCS are likely. Established projects place a strong emphasis on education in end of life care: CMC has trained almost 13 000 and the C&P Project ∼600 users.

### Funding

EPaCCS use has been boosted by incentives like CQUINs (Commissioning for Quality and Innovation), LES and DES (Local Enhanced Services and Directed Enhanced Services) and PDMA (Practice Delivery and Membership Agreement). Established EPaCCS projects have benefited from such payments. They do, however, prioritise other means of fostering engagement to avoid superficial involvement and unsustainable peaks of activity (box 3).

Box 3 **Further drivers used by EPaCCS projects**

- Tapping into congruent projects, initiatives and infrastructure—for example, 2% DES (Directed Enhanced Service) for avoiding unplanned hospital admissions or existing end of life care educational initiatives.
- Representing projects by appealing to the core values of users—improved patient care rather than cost savings; a clinical and patient care project, that is IT-facilitated rather than an IT project.
- The use of sensitive language and broad user involvement in developing patient and public facing informational materials.
- Expansion of the concept—for example, towards ‘Urgent Care Plans’ (CMC) for all patients with complex needs, which increases the scope of the service and reduces the ‘death register’ associations.
- Reliable feedback loops, including investigation of incidents.
- Quality improvement projects using evidence from EPaCCS as baseline and follow-up data.
- Extensive information provision, marketing and awareness raising activities.

## Where next?

### Link to other work streams and reframe

Until recently, EPaCCS were discussed almost exclusively within end of life care contexts. Yet they are only one of numerous initiatives which

1. aim to improve coordination and communication across care settings
2. prioritise patient choice
3. require improved interoperability between information systems
4. use electronic records
5. generate Big Data and
6. seek to shift more care into the community.

EPaCCS need to be better integrated into work streams beyond end of life care. While this may redefine their nature and be experienced as disruptive on both sides, it will make EPaCCS more sustainable, cost-effective and capable of contributing 8 years of development and implementation experience.

Many EPaCCS projects are expanding their scope—for example, towards urgent care plans for frail and elderly patients. This may become a general trend and increase uptake for end of life patients by bypassing anxieties and taboos. The benefits need to be balanced, however, against the danger of diluting the urgency and special needs around the end of life.

### Provide an overview of current EPaCCS and lessons learnt

Detailed descriptions and evaluations of EPaCCS and rigorous broader research are urgently needed. This will enable crucial knowledge exchange and stocktaking. It may also prompt rethinking of the number of EPaCCS needed across a geographical area. Priorities include bringing together outcomes data from currently operational projects; systematically comparing project features and relating them to markers of success, growth and sustainability (see online supplementary table S1, for an initial framework for comparing projects); benchmarking for adequate levels of use; rich descriptions of the contexts in which EPaCCS projects are developed and implemented; analysis of the types of patients on and off EPaCCS and the length of time they are on them; analysis of ‘failed’ projects and bottlenecks for successful projects. Such background work is needed to inform robust study designs, able to detect causal associations and underpin more reliable economic evaluations of EPaCCS.

### Continue work on information standards

An information standard for EPaCCS—SCCI 1580 (previously ISB 1580)—has been available since 2012. Continued work in this direction will facilitate further information sharing at the end of life. Such standards, however, should not be overly prescriptive and allow teams to respond to local needs and culture. In addition, the benefits of increased data capture should be continuously balanced against the (opportunity) costs of increased recording time by staff, in many cases busy clinical staff.

### Rethink national funding

While national funding may seem unlikely in the current climate, decisions need to consider the large number of EPaCCS projects working in isolation, funded separately and to very different degrees. Funding for joint working and knowledge exchange may reduce spending by enabling uptake of existing EPaCCS and sharing hard won lessons. It is important to consider economies of scale. While a more robust comparison of costs is needed, the one made here suggests that the much larger CMC project has come out, proportionately, significantly less costly than the smaller C&P Project.

### Rethink individual and community involvement

Currently, patients can have their EPaCCS record printed out. Expectations and solutions for greater individual and community involvement can only grow. For instance, EPaCCS apps are already under development. As mentioned, there is a growing number of online consumer tools for recording care wishes and preferences. Community development approaches to advance care planning are being experimented with, where people who have been carers act as guides for those undergoing the experience of terminal illness.^52^ In these initiatives, health professionals help identify individuals suitable for such roles and ratify decisions made. The time will come when most individuals will be initiating their own record of end of life care wishes and preferences, informal carers will be updating care summaries and increased community involvement will be the only way to deal with limitations of service capacity. A radical shift in this direction may be needed now.

### Consider the applicability of England's EPaCCS to other national and global contexts

The UK's National Health Service is a tax funded, free at the point of care, developed world healthcare service. The UK has also been ranked first, out of 80 countries, for the quality of its palliative care provision.^53^ The applicability of England's EPaCCS experience to other national and local contexts is therefore a fascinating and challenging question. Rigorous evidence and conceptual and theoretical work on the mechanisms of effecting change is needed before judgements of potential transferability of approach can be made. Numerous factors will need to be articulated and linked in developing a conceptual model of the effectiveness of EPaCCS in the context of England's healthcare system, IT landscape, palliative and end of life care services and skills, professional and public attitudes, etc. In turn, key drivers and challenges will need to be represented at a level of generality that allows comparison with other countries, even if, on the surface, variables differ widely. Paradoxically, England may not be the best place for its own electronic innovation in palliative and end of life care. Countries with a greater maturity of IT systems and HIE tools, more consistently distributed staff IT skills, lighter regulatory frameworks of IG and more centralised decision-making in a local health economy may be better placed to institute electronic coordination systems in palliative and end of life care.

## Conclusions

According to an already dated estimate, there have been 48 reports on palliative and end of life care in the UK since the national End of Life Care Strategy of 2008, demonstrating striking levels of concordance of analysis, agreement on main issues and lack of subsequent progress.^54^ With regard to patient data sharing, a YouGov survey found that 30% of respondents were ‘shocked’, 40% ‘annoyed’ and 61% ‘worried’ that their GP records were not available to accident and emergency departments (8% were ‘not bothered’ and 4% thought that was how it should be).^55^ There is energy and expectation for change in end of life care and data sharing in the UK. Learning from the successes and failures of EPaCCS projects cannot but help connect better the dots, data and people, leading to better care for those approaching the end of their lives.
