# Supplementary material for: Crash course in EPaCCS (Electronic Palliative Care Coordination Systems): 8 years of successes and failures in patient data sharing to learn from
Source: BMJ Support Palliat Care. 2016 Sep 16;8(4):447–55. doi: 10.1136/bmjspcare-2015-001059 (PMC6287568; doi:10.1136/bmjspcare-2015-001059)
Supplement: Supplementary tables [file bmjspcare-2015-001059supp001.pdf]

Table 1: Comparison of the features and context of Coordinate My Care, the South West EPaCCS, and the Cambridgeshire and Peterborough Project for Data Sharing in End of Life Care (last updated Jun 2016)

Parameter	Coordinate My Care	South West EPaCCS	Cambridgeshire & Peterborough
**Area covered**			
**Clinical Commissioning Groups**	33	9	1
**Population (m)***	9.27	3.96	0.86
**GP practices**	1,887	535	107
**Acute Trusts**	22	16	4
**111 services†**	4	2	1
**Ambulance Services‡**	2	1	1
**GP Out of Hours Services§**	12	6	2
**Hospices****	16	8	3
**Care homes††**	2,516	>882	177
**District nursing teams‡‡**	19	> 104	6
**Palliative care teams§§**	40	>24	8
**Other known settings/teams where access desirable (e.g. community hospitals)**			5
**Key settings/ teams where access desirable** *(excludes care homes and 'other'; counts 5 main teams/ settings in acute trusts)****	2,090	>760	153
**Total of settings/ teams where access desirable**	>4,606	>1,642	335

* Data on the population of all three areas and on GP practices covered by the South West EPaCCS are from the CCG Directory at http://www.england.nhs.uk/resources/ccg-directory/ (Mar 2013 update). Data on GP practices in the *CMC* and the C&P areas are from the service development teams (2015).

† 111 services providers: London Ambulance Service NHS Trust, Care UK, London Central & West Unscheduled Care Collaborative, and Partnership of East London Co-operatives in the *CMC* area; Care UK and South Western Ambulance Service NHS Foundation Trust (South West); Herts Urgent Care (C&P).

‡ London Ambulance Service NHS Trust and South East Coast Ambulance Service NHS Foundation Trust (*CMC* area), South Western Ambulance Service NHS Foundation Trust (South West) and East of England Ambulance Service NHS Trust (C&P).

§ Counts are for services and not sites/bases, which can be a significant number (e.g. BrisDoc have 7 bases). Information for the South West from individual CCGs. Information for *CMC* from project team.

** Numbers are for hospices as organisations rather than sites. Information was obtained from the project leads.

†† Numbers for C&P obtained from carehome.co.uk. Numbers for the South West from individual CCGs, 1 unable to provide data. Numbers for *CMC* from project team.

‡‡ Numbers for the South West obtained from individual CCGs, 2 unable to provide data. In the 7 CCG that provided data, team numbers varied from 1 (Bath and North East Somerset) to 41 (Somerset). Number for C&P includes 4 core DN teams and 2 specialist DN teams (Respiratory and Cardiovascular) seen as likely to be highly involved in end of life care. *CMC* data obtained from project team.

§§ Numbers for the South West obtained from individual CCGs, 2 unable to provide data. In the 7 CCG that provided data, team numbers varied from 1 (Gloucestershire CCG) to 5 (North Somerset and Somerset). There is a potential for teams covering more than one CCG being counted twice. Data for *CMC* and C&P obtained from the project teams.

*** A&E, Medicine for the Elderly, Acute Oncology, Respiratory Conditions and Cardiovascular Conditions. Hospital palliative care teams are counted, whenever possible, under Palliative care teams.

Parameter	Coordinate My Care	South West EPaCCS	Cambridgeshire & Peterborough
**Baseline EoLC statistics for area (2011-13)†††**	**London all (see below for *CMC* only)**	**South West all (see below for EPaCCS only)**	**C&P all**
**Deaths in hospital**	55.07%	43.46%	42.29%
**Deaths at home**	21.34%	22.99%	27.08%
**Deaths in care homes**	14.75%	26.10 %	22.44%
**Deaths in hospice**	6.53%	5.25%	5.47%
**Key evidence of impact**			
**% of deaths in preferred place**	*Aug 2010 – May 2016 data* for 7,614 patients<br>71.8% – first preferred place<br>5.9% – second preferred place		
**% deaths at home**	36.2%		
**% deaths in hospital**	17.6%	*Study of 3,012 EPaCCS patients and over 67,000 total deaths‡‡‡*<br>EPaCCS patients / Non-EPaCCS patients<br>cancer **9.8%** **33.9%**<br>other **8.3%** **49.4%**	
**Impact on ambulance dispatch**	50% lower for *CMC* patients§§§		
**Impact on conveying patients to emergency department**	80% less likely for *CMC* patients		
**Impact on hospital admissions**	Data not yet available		
**Estimate of cost savings per patient**	£2,100****		

††† Place of death data taken from the CCG End of Life Care profiles provided by the National End of Life Care Intelligence Network using Office for National Statistics data, see http://www.endoflifecare-intelligence.org.uk/profiles/CCGs/Place_of_Death/atlas.html. Figures for the *CMC* and South West EPaCCS areas were obtained by adding raw numbers for the CCGs covered by them and estimating average percentages. C&P is a single CCG, so data were taken directly from the atlas. Figures for *CMC* and the South West area include EPaCCS patients who have died. The C&P Project is newer than the latest available place of death data so EPaCCS patients are not included. See *Key evidence of impact* for EPaCCS-specific place of death data.

‡‡‡ Whole Systems Partnership for NHS Improving Quality (2013). *Economic Evaluation of the Electronic Palliative Care Coordination System Early Implementer Sites*. http://thewholesystem.co.uk/wp-content/uploads/2014/07/economic-eval-epaccs.pdf

§§§ Data on ambulance dispatch and emergency department referrals are from a 111 Learning Programme Evaluation in collaboration with *CMC.*

**** National Information Board (2014). *Personalised Health and Care 2020. Using Data and Technology to Transform Outcomes for Patients and Citizens. A Framework for Action*. https://www.gov.uk/government/uploads/system/uploads/attachment_data/file/384650/NIB_Report.pdf.

Parameter	Coordinate My Care	South West EPaCCS	Cambridgeshire & Peterborough
**IT platform, interface and usability**			
**Broad type of solution**	Standalone web-based register	6 standalone web-based registers, using the same solution and adapted to fit with local structures and processes	Templates (for data entry) in all four GP record keeping systems; View (for recipients) in the dominant local system; easy-to-use processes of data sharing in the dominant local system and more complex workarounds in the other systems
**IT system and services provider**	HealthShare (Intersystems)	Adastra	SystmOne for data sharing<br>SharePoint and Excel Power Pivot (platforms) and Ascribe and CCG IT team (developers) for service monitoring and reporting solutions – see below in this column on "Dashboards"
**Manual data entry – automatic population balance**	Primarily manual data entry. Automatic extraction of demographics from PDS (the Personal Demographics Service, part of the Spine[††††]). PDS facilities available without smartcard since Nov 2015	Manual data entry	SystmOne users: automatic population from and into patient record + manual data entry<br>Other IT systems users (in GP practices):<br>- automatic population from and into patient record + manual data entry, from which an attachment document generated and then uploaded onto SystmOne record, usually by an MDT coordinator<br>- manual data entry on SystmOne record by MDT coordinators
**Settings/teams in which in-principle access**	All, including in settings without N3[‡‡‡‡] (additional authentication needed)	All, provided N3 access	Users of SystmOne or SystmOne Clinical Records Viewer which locally are (Apr 15):<br>72% (77) of GP practices<br>95% (19) of urgent and acute services[§§§§]<br>100% (17) of community and specialist teams and settings[*****]<br>No care home

[††††] The Spine is "a collection of national applications, systems and directories that support the NHS in the exchange of information across national and local systems. Hosts demographic information for 80 million citizens plus a number of national applications including Summary Care Record and the Electronic Prescription Service" (http://systems.hscic.gov.uk/ddc).

[‡‡‡‡] The NHS private data network

[§§§§] Estimated as follows: one 111 provider; 1 Ambulance Service; 2 OOH services; 3 multi-profile acute hospitals with 5 key wards with a significant role in end of life care counted within each (A&E, Medicine for the Elderly, Acute Oncology, Cardiovascular, Respiratory) and 1 hospital specialising in heart conditions (the only one with no access).

[*****] Estimated as: 6 DN teams (4 area teams, Cardiovascular and Respiratory), 8 specialist teams and 3 hospices.

Parameter	Coordinate My Care	South West EPaCCS	Cambridgeshire & Peterborough
**Settings/teams in which access in own and intensely used record keeping system**	None	Some (Adastra practices and OOH services), but separate application	Some of the SystmOne users (Apr 15):<br>72% (77) of GP practices<br>20% (4) of urgent and acute care settings<br>12% (2) of community and specialist teams
**Types of patient information templates**	Generic Personalised Urgent Care template, originally with an end of life care focus	Generic end of life care	Generic end of life care
**Practice/ GSF register functionalities** (screens for all end of life care patients in a practice)	Yes	Yes	A separate Clinical Dashboard, under pilot – a SharePoint solution
**Auditing and reporting functionalities available to GP practices, LCGs, CCGs or other organisations**	Audit and management information reports can be requested ad hoc or subscribed to on a monthly basis (currently >100 subscriptions). Minimal reporting functionality currently available to end users	Minimum uptake of intended approach, where reports were expected to be run by the system host (e.g. CCG), used for service improvement and also fed back to practices, see below for new solution	A Business Intelligence Dashboard is under development – Excel Power Pivot and SharePoint solution developed by CCG IT team and Serco
**Auditing and reporting functionalities available to project development team**	Yes, via dedicated data warehouse/business intelligence solution	Currently 'no' and considered a serious obstacle. New reporting solution hosted by project team is developed in collaboration with Adastra and the National End of Life Care Intelligence Network	Currently incomplete (the Business Intelligence Dashboard will also be available to project team) and considered a serious obstacle
**Set-up requirements and processes**			
**System requirements, including security**	Normally, N3 connection (non-N3 connectivity via *CMC* two-factor authentication solution)<br>Password protection	N3 connection<br>Password protection	SystmOne or SystmOne Clinical Records Viewer<br>Smart card for each user
**Requirements for access rights of individuals**	Legitimate relationship with patient<br>Authorised by manager<br>Self-certified to have adequate knowledge to use *CMC* safely and as appropriate to role<br>Completed *CMC* training<br>Completed NHS IG training<br>Signed *CMC* Acceptable Use Policy	Legitimate relationship with patient/ authorised by manager	Legitimate relationship with patient/ authorised by manager
**Requirements for access rights of organisations**	Suitable attainment on the Health & Social Care Information Centre IG Toolkit<br>Signed *CMC* Joint Data Controller Information Sharing Agreement		No agreements further to standard SystmOne use across organisations<br>Information sharing agreement signed for Dashboard development and use

Parameter	Coordinate My Care	South West EPaCCS	Cambridgeshire & Peterborough
**Routine use processes and workflows**			
**Principles of patient inclusion**	Expanded from end of life care plan to an urgent care plan	End of life care only	End of life care only
**Explicitness of consent/ awareness of end of life status**	Not necessarily, as non end of life patients can be consented for the creation and sharing of an urgent care record	Likely, as Patient Information Leaflet open about end of life care, but not necessarily so – can be consent for data sharing only	Not necessarily – can be consent for data sharing only
**Financial incentives**	Variable, currently linked to 2% DES[†††††]		Variable, in 2015 linked to 2% DES in all Local Commissioning Groups (LCGs) and PDMAs[‡‡‡‡‡] in 3 LCGs
**Training and awareness raising**			
**No of staff trained**	12,896 (May 2016) including e-Learning and awareness, refresher, and super-user training	Difficult to estimate	596 (Jan 2015)
**Dedicated website**	http://coordinatemycare.co.uk/	No	Key information (documents embedded in template) stored on local Primary Care Information Team website
**User support**	Telephone and email advice line	Clinical Lead	Clinical Lead, Project Manager, Educational Facilitators and IT experts
**Ongoing awareness raising activities**	Persistent networking, lobbying and presentations<br>Extensive set of information materials<br>High profile presence in policy reports, the media and professional journals<br>GP and Patient forums	Persistent networking, lobbying and presentations<br>Quality improvement workshops	Persistent networking, lobbying and presentations<br>Paper mailshots to all local GPs, practice managers and DN teams<br>Dissemination of research outputs
**Uptake of system**			
**Total number of records**	29,083 *(Aug 2010 – May 2016)*	Unknown, no reporting functionalities available to project team	Unknown, no reporting functionalities available to project team
**Records in last 12 months**	7,502 *(June 2015 – May 2016)*		
**Record creation by user group**	*Aug 2010 – May 2016*<br>34% – hospice teams		Anecdotal evidence for one of the specialist teams and GPs creating the majority of summaries/ plans

[†††††] 2% DES – Directed Enhanced Service for avoiding unplanned admissions
[‡‡‡‡‡] Practice Delivery and Membership Agreement incentive

Parameter	Coordinate My Care	South West EPaCCS	Cambridgeshire & Peterborough
**Record creation by user group, cont.**	28% – GPs<br>26% – acute services<br>12% – community services		
**Patient groups**	*Aug 2010 – May 2016*<br><br>52% cancer<br>18% dementia<br>7% cardiac<br>4% neurological<br>6% respiratory<br>2% renal		
**Use in CCGs**<br>***High** – ≥ 80% of expected EoLC population on register*<br>***Advanced** – 30÷79%*<br>***Moderate** – 10-29%*<br>***Limited** – ≤ 9%*	*1 Jun 2015 – 31 May 2016*<br><br>Advanced – 12.1% (4 CCGs)<br>Moderate – 45.5% (15 CCGs)<br>Limited – 42.4% (14 CCGs)		
**Use in GP practices**	28% of all records on *CMC* created in GP practices, *Aug 2010 – May 2016*<br><br>12,215 views by other than creating organisation (13.99% of existing *CMC* records) in GP practices, *Apr 2013 – May 2016*		
**Use in urgent and acute care settings**	26% of all records on *CMC* created in acute settings, *Aug 2010 – May 2016*<br><br>Views of existing *CMC* records by other than creating organisation, *Apr 2013 – May 2016*<br><br>Acute – 6,648 (8.26% of existing *CMC* records)<br>111 – 13,031 (13.75%)<br>A&E – 31 (0.06%)<br>Ambulance – 8,239 (10.31%)<br>Out of hours – 5,308 (8.27%)		
**Use in community and specialist teams**	Percentage of all records created, *Aug 2010 – May 2016*<br>Hospice teams – 34%<br>Community teams – 12%		

Parameter	Coordinate My Care	South West EPaCCS	Cambridgeshire & Peterborough
**Use in community and specialist teams, cont.**	Views of existing *CMC* records by other than creating organisation, *Apr 2013 – May 2016*<br>Hospice teams – 10,808 (10.71% of existing *CMC* records)<br>Community teams – 3,858 (4.58%)		
**Cost and timelines**			
**Total**	£1.5 m (as of Apr 14)	No pooled estimates available	£245,000
**IT solution**		£9,000 per PCT for set up + 2p per head (the whole population of an area) per annum	No cost for SystmOne-based data sharing solution; estimate of £50,000 for Dashboard development
**Project initiation date**		2008	Jul 2012
**Go live date**	Aug 2010		Feb 2014
**Project team and setting**			
**Host organisation**	Royal Marsden NHS Foundation Trust	The individual CCGs + Devon Docs in one of the areas	Has had three hosts: Urgent Care Cambridgeshire, Cambridgeshire Community Services NHS Trust and Cambridgeshire & Peterborough NHS Foundation Trust (current)
**Members of staff, development phase**		3	8-12
**Members of staff, expansion and maintenance phase**	19	1	8
**Project lead**	Dr Julia Riley	Dr Julian Abel	Dr Stephen Barclay, Clinical Lead<br>Ian Merrick, Project Manager
**Sustainability work**			
	Even fuller transformation into a system for Urgent Care Plans as opposed to end of life only. Extensive interoperability roadmap. Close alignment with NHS England's urgent and emergency care technology strategy. Patient/carer access roadmap drawn up, including initiation of urgent care planning process by patient. Strong Clinical Quality focus, including regular urgent care service feedback and well-defined data quality reporting and remediation processes.	Stronger integration with quality improvement initiatives to encourage routine use and action on feedback.	Even fuller integration with routine record keeping and local IT solutions for data sharing.

Tables 2a and b: Data fields in the 3 EPaCCS

Table 2a: Data fields in the 3 EPaCCS in relation to the National Information Standard (SCCI1580)

The latest version of the standard is from Sep 2015:
http://www.hscic.gov.uk/media/18510/1580112015spec/pdf/1580112015spec.pdf

Fields marked by ***** in the first column are mandatory for being *present* in an EPaCCS as per SCCI 1580. Whether *completion* is made mandatory is project-specific.
√ - the field is included in the respective EPaCCS
√ - the field is also mandatory
√ - the field is automatically populated. In the case of *CMC*, the information is taken from the Spine. In the case of the C&P Project, the information is taken from the patient's GP practice record.
√ - the field is automatically populated, but information may be missing often as it is not core information in a patient's record.
~√ - no separate field is available, but there is a strong message in Guidance Notes and/or training sessions to add this type of information in a free text field.

For the C&P Project, the table has been completed for the SystmOne Template, which is the richest of the four templates (also in EMIS Web, EMIS LV and Vision), and for the SystmOne View. Part of the automatically populated fields is visible only in the View (for recipients, mostly in out of hours and acute settings) and not on the Template (generally filled in by GPs, specialist nurses and district nurses).

Field as per SCCI 1580	CMC	SW	C&P
**1. Consent status***	**√**	**√**	√
**2. Record creation* date AND record amendment* dates**	√	√	√
**3. Plan and requested actions**			
**Planned review date**	**√**	**√**	√
**Cardiopulmonary resuscitation decision**			
Whether a decision has been made and the decision	**√**	√	√
Date of decision	√	√	√
Location of the documentation	√		√
Date for review	√		
**4. Person demographics**			
Name*	**√**	**√**	**√**
Preferred name	√		
Date of birth*	**√**	**√**	√
Address*	**√**	**√**	√
NHS number*	**√**	√	√
NHS number status indicator code*	√		
Telephone number	**√**	√	√
Gender	**√**	**√**	√
Relevant contacts	√	√	√
Main carer name and contact details	**√**	√	√
Availability of carer support*	√	√	√
**5. Special requirements**			
Need for interpreter			
Preferred spoken language	√		
Functional status	√		
Disability	√		

Field as per SCCI 1580	CMC	SW	C&P
End of Life Care tools in use, e.g. Gold Standards Framework	√	√	√
**6. Information and advice given**			
Is main carer aware of person's prognosis?	√	√	√
Is person aware of the cardiopulmonary resuscitation clinical decision?	√	√	
Family member/carer informed of cardiopulmonary resuscitation clinical decision?	√	√	
**7. GP practice**			
GP name*	√	**√**	√
GP practice details*	**√**	√	√
**8. Key worker**			
Name	√	√	√
Telephone number	√	√	√
**9. Services and care**			
Name of health and social care staff and professionals involved in care	√	√	√
Professional group	√	√	√
Telephone number	√	√	√
**10. Diagnoses**			
Primary end of life care diagnosis*	**√**	√	√
Other relevant end of life care diagnoses and clinical issues	√	√	√
Likely prognosis	√		√
**11. Allergies or adverse reactions**	√		√
**12. Medications and medical devices**			
Whether a 'just in case box' or anticipatory medicines have been prescribed	√	√	√
Where these medicines are kept	√		~√
**13. Legal information**			
**Advance statement**			
Requests or preferences that have been stated	√	√	√
**Advance Decision to Refuse Treatment (ADRT)**			
Whether a decision has been made and the decision	√	√	√
Date of decision		√	
Location of the documentation	√		~√
**Lasting Power of Attorney or similar**			
Name and contact details of person appointed with Lasting Power of Attorney (LPA) for personal welfare	√	√	√
- without authority to make life-sustaining decisions			
- with authority to make life-sustaining decisions			
**14. Person and carers' concerns, expectations and wishes**			
Preferred place of death, 1st and 2nd choice if made	√	√	√
Names and contact details of others (1 and 2) that the person wants to be involved in decisions about their care	√	√	√
Other relevant issues or preferences around provision of care?	√	√	√
**15. Actual place of death**	√	√	√
**16. Date of death**	√	√	√

Table 2b: Further fields in the 3 EPaCCS

This table presents further fields available in the 3 EPaCCS, in addition to the ones specified under the national information standard. In some cases the additional categories extend the SCCI requirements; in others they specify the generic SCCI categories (e.g. “Services and care” or what “Other relevant issues and preferences”, see 9 and 14 above).

Broad category of further information	CMC	SW	C&P
**Personalisation of care plan and advance care planning**	Ceiling of treatment<br>Anticipated problems and symptom management	Advance care planning document – given, read and completed<br>Preference for continuation/ discontinuation of hospital treatment<br>Preference for active follow-up or not	Current coordinated support plan<br>Anticipatory care plan
**Medication**	Medications list for Urgent Care Services (drop-down menu)<br>Other medications information (free text)		Current medication (acutes and repeats)<br>Prescribing tool – lists commonly prescribed palliative drugs and suggested starting doses; links to prescription printing facilities
**Diagnostic and measurement tools**	iPOS (integrated Palliative care Outcome Scale)		
**Other carers and care settings (key contacts and relationships)**	Next of kin and other personal contacts<br>Health and social care contacts	Known to hospice	Macmillan Nurse<br>District Nurse<br>Community Matron
**Social aspects of care**	Social care package<br>Equipment, help with home care, support from family members<br>DS1500	DS1500	DS1500<br>Housing details (e.g. lives alone)<br>Religion
**After death – planning**		Will GP sign death certificate?	
**After death – review**	Reason for variance from preferred place of death, where relevant	Reason for variance between preferences/ care plan and actual death	Reason for variance between preferences/ care plan and actual death<br>Team has considered bereavements support<br>Team has reviewed patient's EoLC after death
**Incentives-related fields**	No specific fields, but *CMC* uses its reporting service, including data quality measures, to provide incentives information as required by commissioners		2 fields supporting the Quality and Outcomes Framework data collection (on practice register and MDT review)<br>5 fields supporting admission avoidance DES<br>1 field for an advance care planning incentive in 2 LCGs

Broad category of further information	CMC	SW	C&P
**Web links to forms and information leaflets**	Custom user interface with external web links wherever appropriate, e.g. re ADRT, DNACPR		Information leaflet for patients and carers<br>DNACPR form<br>Advance Decision to Refuse Treatment (ADRT) form<br>Referral forms to local specialist teams and hospices<br>Prescribing chart and guidance<br>Leaflet for carers on final days of life<br>Lasting Power of Attorney (LPA) information<br>Cremation forms<br>Bereavement supports services information<br>Specification for Avoiding Unplanned Admissions enhanced service
**Electronic referral forms**			To community nursing services and one of the local specialist palliative care teams
**Local pathways for transfer of information**	Existence of *CMC* care plan is flagged on urgent care services' systems (automated or manual process)<br>*CMC* patient telephone number list sent daily to 111 London call routing service, allowing *CMC* patients to be handled by *CMC*-aware call handlers even at periods of high demand		Special Patient Note prompt – locally standardised format for 111
**Free text fields**	Yes	Yes	Yes
